# Tibial Nerve Palsy: An Atypical Presentation of a Popliteal Cyst

**DOI:** 10.7759/cureus.27984

**Published:** 2022-08-14

**Authors:** Nikolaos Stefanou, Georgios Kalifis, Theodorakys Marin Fermin, Antonios Koutalos, Vasileios Akrivos, Zoe Dailiana, Sokratis Varitimidis

**Affiliations:** 1 Department of Orthopedic Surgery and Musculoskeletal Trauma, University Hospital of Larissa, Larissa, GRC; 2 Department of Trauma and Orthopedics, Hull University Teaching Hospitals NHS Trust, Hull, GBR; 3 Department of Trauma and Orthopedics, Aspetar Orthopedic and Sports Medicine Hospital, Doha, QAT

**Keywords:** arthroscopy, knee, tibial nerve palsy, baker's cyst, popliteal cyst

## Abstract

Popliteal cysts represent one of the commonest knee pathologies in the adult population. The vast majority of cases may be treated conservatively as symptoms tend to resolve spontaneously. However, few patients may experience persistent pain and nerve-related symptoms not responding to conservative management. We present a case of a 46-year-old patient who suffered from tibial nerve palsy due to a popliteal cyst that was treated successfully with combined open and arthroscopic procedure. It is important to understand that popliteal cysts rarely may lead to tibial nerve entrapment and in selected cases operative management may be indicated.

## Introduction

Popliteal cysts (Baker cysts) constitute the most common cystic lesions around the knee joint in adults [1]. Cystic lesions around the knee can be classified into primary, not associated with intra-articular pathology, and secondary, often associated with concomitant intra-articular lesions, especially meniscal tears [2]. The bursae of the medial gastrocnemius head and the semimembranosus have been reported to communicate with the knee joint, causing a valve-like mechanism in the posteromedial aspect of the knee, and in the presence of excessive synovial fluid production, one-way flow may lead to popliteal cyst formation and gradual enlargement [3]. Clinical presentation often includes knee pain and swelling in the posterior aspect of the knee and can mimic deep vein thrombosis (DVT) [4]. Often patients respond to conservative management, however, in selected cases with large size of the cyst that affects the range of motion of the knee joint and persistent or aggravating symptoms, operative management is indicated [5].

Here, we present a case of a popliteal cyst and tibial nerve irritation symptoms that were successfully treated with a combination of open excision and arthroscopic meniscectomy after the initial failure of conservative treatment.

## Case presentation

A 46-year-old male, working as a heavy laborer, was admitted to our department with recurrent, deteriorating vague right knee pain and a palpable mass in the popliteal fossa for the last six months as well as lower leg numbness and calf muscle atrophy, that was noticed for the first time one month earlier. He also described "locking" episodes of right knee that have been spontaneously resolved. The patient denied a history of injury or any other symptoms referring to a systemic disease, such as fever, fatigue, or weight loss. The patient was able to do unaided full weight bearing, however, he was frustrated as he subjectively considered his condition to have deteriorated (pain, functional impairment, and palpable enlargement of the mass).

Before presentation to our clinic, the patient had been managed conservatively, without success, after the initial diagnosis by ultrasound. Physiotherapy, non-steroid anti-inflammatory drugs (NSAIDs), and aspiration plus 40 mg of triamcinolone acetonide injection (x1) failed to address symptoms. Physical examination revealed a moderate localized swelling of the posterior aspect of the right knee that was tender on palpation. Active range of motion (ROM) was 15°-120° degrees and passive ROM was 0°-135°. Medial joint line tenderness during palpation as well as positive Appley’s and McMurray’s tests were present. Foucher’s sign (cyst tends to disappear or to decrease with 45° of knee flexion) was positive. Sensory deficit (hypoesthesia) in the distribution of the tibial nerve was present during resting at night or daily activities, and other symptoms like dull pain, numbness, tingling, and burning sensation were referred at the posterolateral side of the leg and the plantar side of the foot, especially in work and deep bending of the knee. No lower leg muscle power weakness was present even though the perimeter of the affected calf was measured more than 2 cm less than the asymptomatic side. Dorsalis pedis and posterior tibialis pulse were present, arterial pressure index (API) was >0.9 and capillary refill time was less than 2 s. No other significant clinical findings were noted.

**Figure 1:**
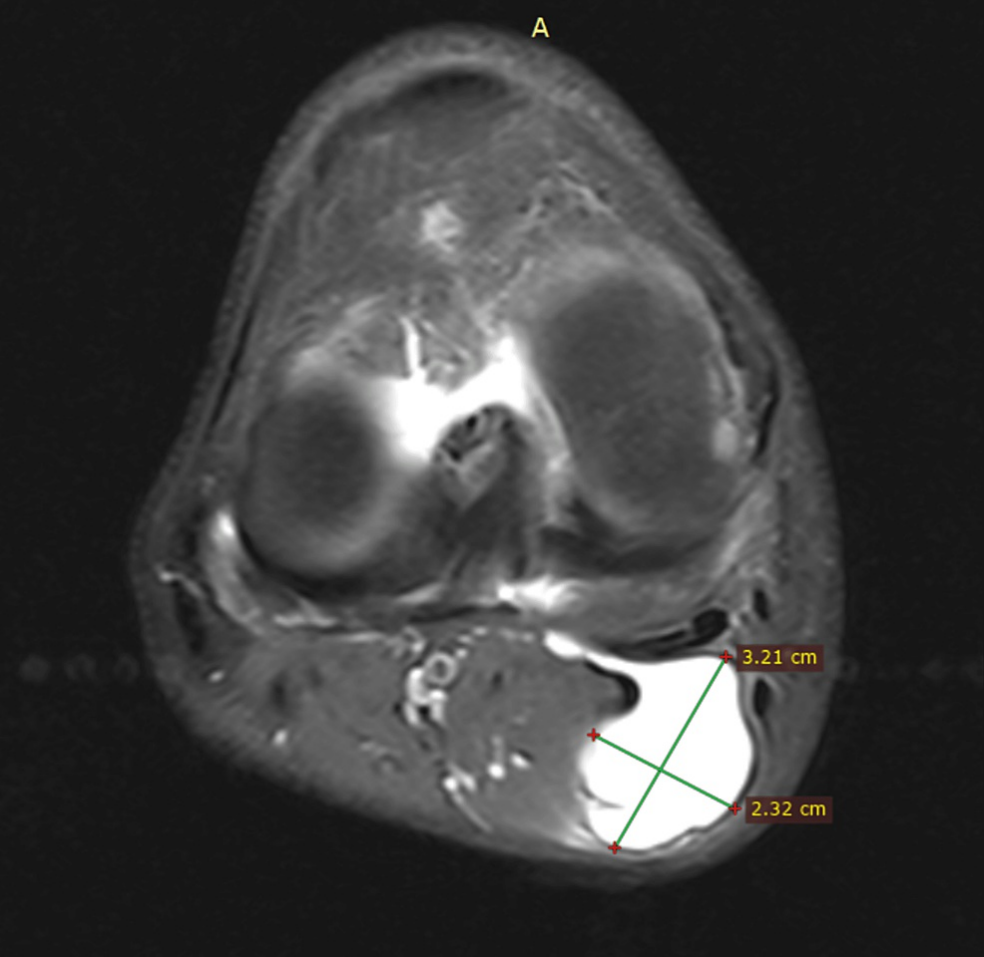
Axial T2-weighted image that shows the pedicle of the cyst adherent to posterior capsule. Selected sample MRI studies demonstrate the size of the cyst in relation to adjacent anatomical structures.

**Figure 2:**
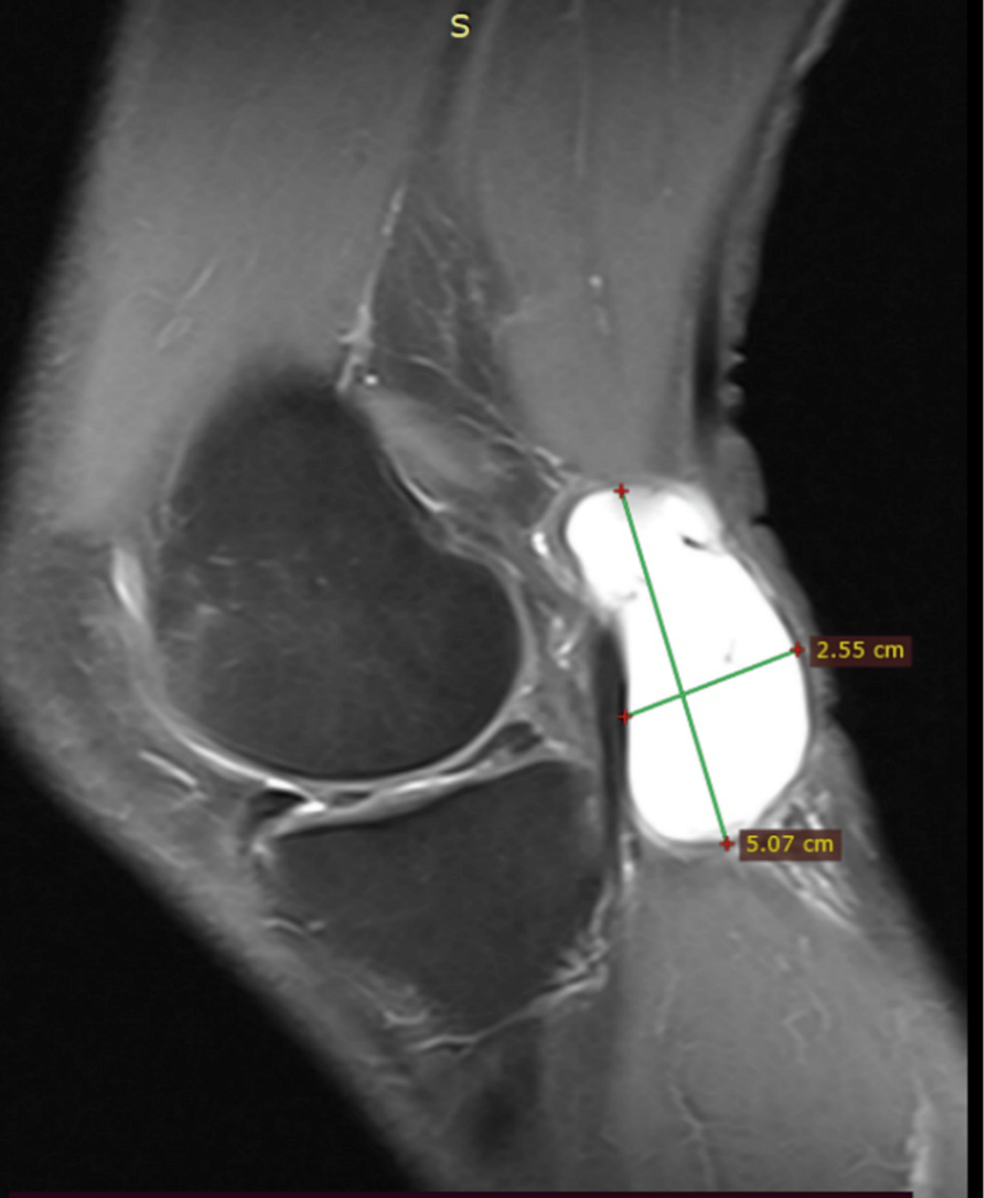
Sagittal T2-weighted image with increased signal of the cyst lying just lateral to the semimembranosus tendon in the popliteal fossa. Selected sample MRI studies demonstrate the size of the cyst in relation to adjacent anatomical structures.

**Figure 3:**
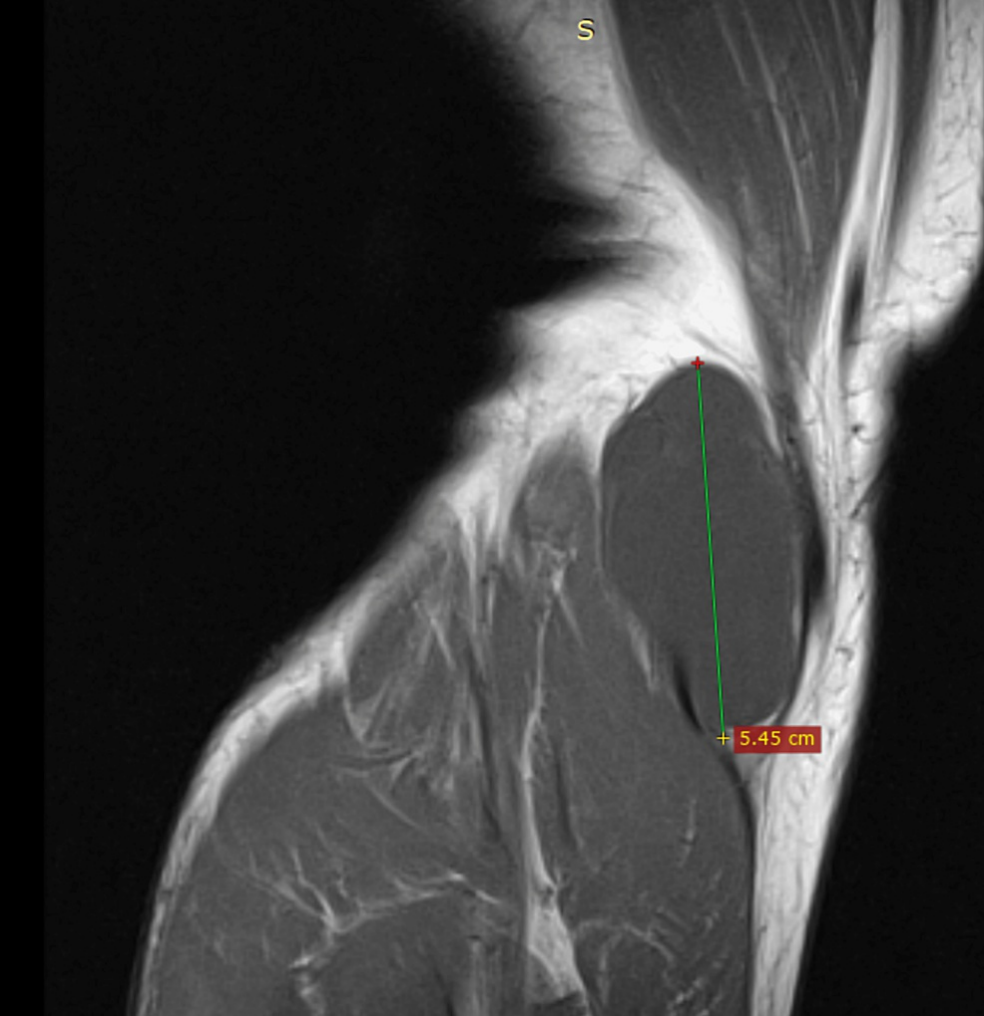
Coronal T2-weighted image that shows a low-intensity signal cyst located near the gastrocnemius medial head. Selected sample MRI studies demonstrate the length of the cyst in relation to adjacent anatomical structures.

Plain radiographs of the right knee showed moderate degenerative changes and non-specific posterior soft tissue swelling. MRI demonstrated a 3.1x5.1x2.5 cm cystic lesion, without direct compression of the tibial nerve, increased signal on T2 and fat suppression sequences as well as T1 low-intensity signal (Figures 1-3). It was located between the semimembranosus tendon and the medial head of the gastrocnemius muscle with a fluid-filled neck of the cyst communicating with the joint (axial images). In addition, a horizontal tear of the posterior horn of the medial meniscus, with characteristics of degeneration, was revealed. No further pathology of the right knee was noted. Blood tests including complete blood count (CBC), c-reactive protein (CRP), and erythrocyte sedimentation rate (ESR) were non-significant.

**Figure 4:**
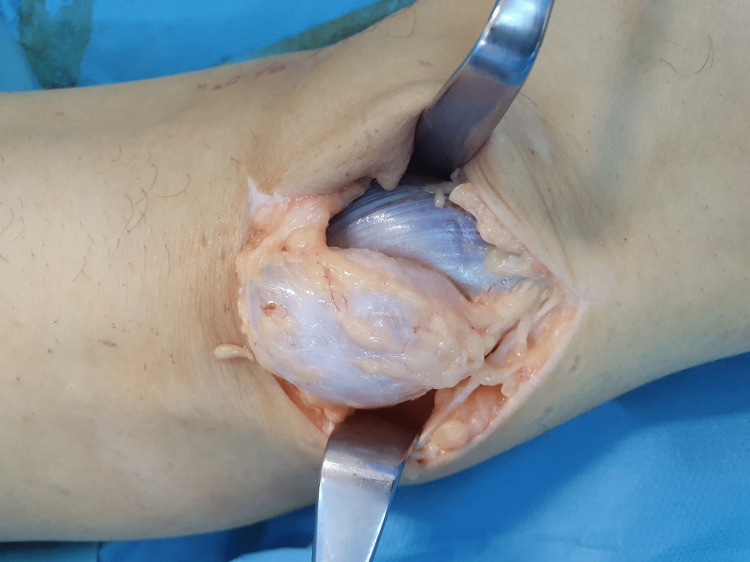
Longitudinal division of the fascia was performed to expose a large cystic lesion arising from the interval between the semimembranosus tendon and medial head of the gastrocnemius, lying superficially over the medial head of the gastrocnemius. Surgical exposure of the cyst under the flexor crease in popliteal fossa.

**Figure 5:**
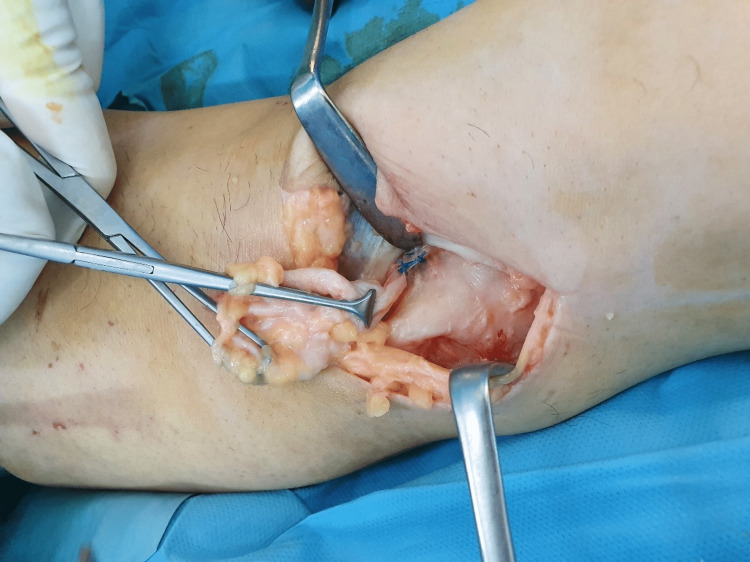
The base of the cyst which was attached to the capsule and communicated with the knee joint was ligated and the cyst was excised. Surgical exposure of the cyst under the flexor crease in popliteal fossa.

**Figure 6:**
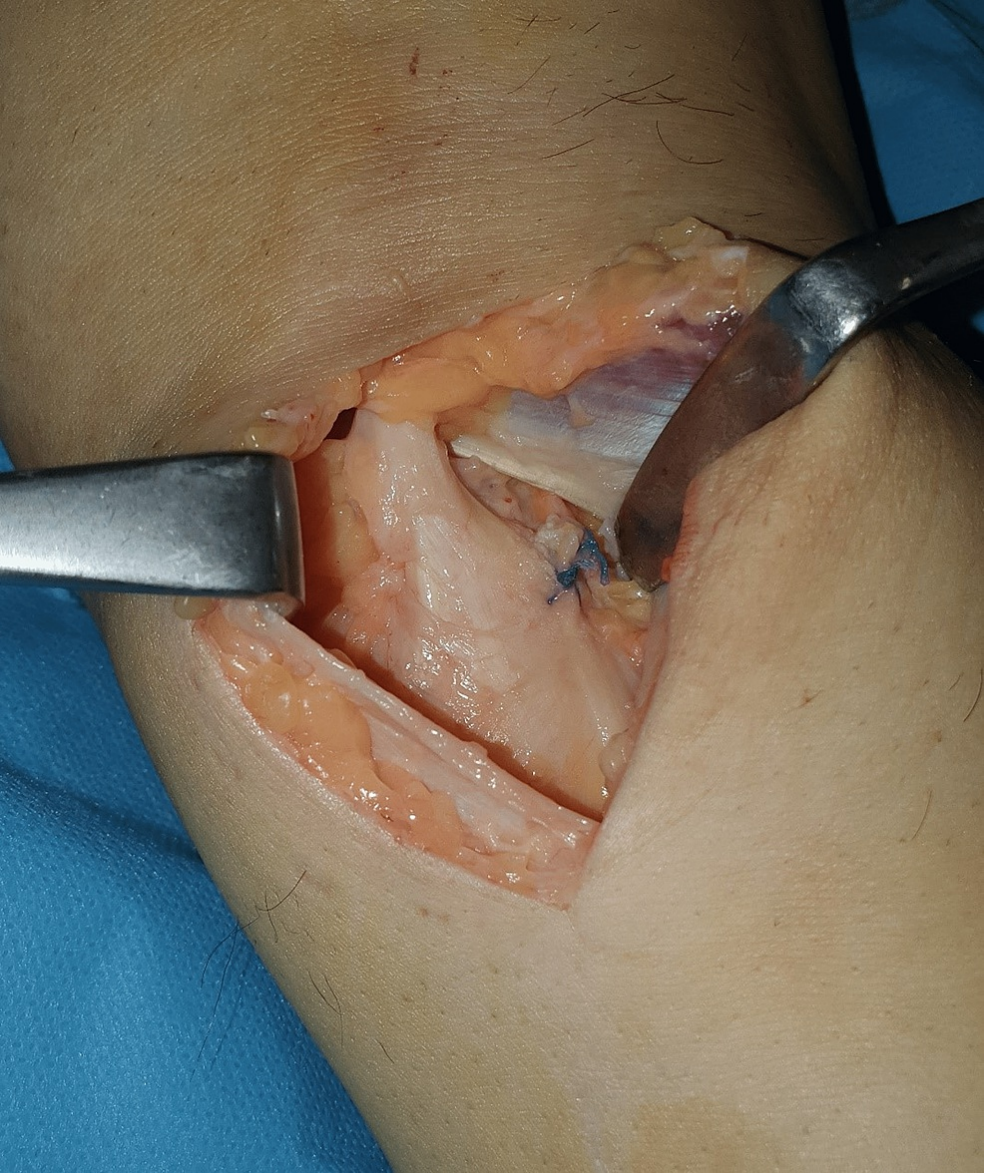
Posterior capsule reinforcement by suturing a plicated pedicle of the semimembranosus tendon, with a number 1 Vicryl suture, was performed. Surgical exposure of the cyst under the flexor crease in popliteal fossa.

After the failure of conservative management, the patient consented to operative treatment offered by our team. A combination of arthroscopic partial medial meniscectomy accompanied by an open excision of the cyst through a posteromedial knee approach was performed. Αs our preferred treatment option is open cyst removal, initially, the patient was placed in a prone position and an “L-shaped” posteromedial incision of the popliteal area was carried out (the long dimension of it over the flexor crease). A longitudinal dissection of the fascia was performed to expose a large cystic lesion arising from the interval between the semimembranosus tendon and the medial head of the gastrocnemius, lying superficially over the medial head of the gastrocnemius and without direct contact with the tibial nerve. The lesion was carefully marked and released by blunt dissection down to its line of attachment to the popliteal space. The base of the cyst which was attached to the capsule and synovium, and communicated with the knee joint, was ligated and then the cyst was excised. The wound was thoroughly irrigated and subsequently the posterior capsule was repaired. The repair was reinforced by suturing a plicated pedicle of the semimembranosus tendon, with a number 1 Vicryl suture (Figures 4-6). After closure by layers with a 2-0 Vicryl suture for the subcutaneous tissue and a Monocryl 3-0 suture for the skin, the patient was placed in the supine position for knee arthroscopy. Further skin preparation and draping were done. Diagnostic arthroscopy by standardized approach using anterolateral (AL) and anteromedial (AM) portals revealed a degenerative cleavage tear of the posterior horn of the medial meniscus and an arthroscopic partial meniscectomy was performed.

Postoperative rehabilitation protocol included weight bearing as tolerated with crutches, in a knee immobilizer locked in extension for one week, followed by removal of the knee immobilizer and progressing to full weight bearing without active and passive ROM restrictions, but assisted by crutches for balance until third postoperative week. Between three and six weeks the patient was allowed to follow a strengthening protocol of the muscles around the knee and return to work was permitted after six weeks. The patient achieved full range of motion without residual pain at the end of the fourth week of the rehabilitation program and sports participation was allowed three months postoperatively. Additionally, sensory symptoms of the tibial nerve had been resolved since the first postoperative week. At six- and 12-month follow-ups the patient regained full and painless ROM.

## Discussion

Popliteal cysts were first described by Robert Adams in 1840 and thoroughly studied by William Baker in 1877; named after the latter, they are the most common cystic lesions around the knee and represent 10-41% of posterior knee masses [6,7]. Anatomically, these cysts correspond to the junction of the gastrocnemius-semimembranosus intermuscular bursae and the knee joint cavity by a one-way bridging valve [8-11]. Popliteal cysts are often asymptomatic and are incidentally found in imaging studies [5,12]. However, the enlargement of such cystic lesions may cause irritating compression of the adjacent neurovascular bundle, in which the tibial nerve is the most vulnerable among them because of its medial and superficial location [9,12,13].

Popliteal Baker's cysts have been thoroughly associated with intra-articular disease in up to 94% of the cases. The medial meniscus posterior horn tears are the most relevant and can be considered an indirect sign of this disease [5]. In the present case report, the patient's persisting pain, range of motion limitation, and rapid progressive numbness onset over the tibial nerve territory were the main complaints. Additionally, the patient presented the characteristic Foucher's sign and MRI findings, including cystic mass and concomitant medial meniscus posterior horn degeneration, which guided the diagnosis, especially in the absence of systemic disease [6,7,9].

It is essential to highlight that the differential diagnosis of popliteal masses includes other intra-articular and extra-articular masses, such as soft tissue, vascular, lymphoid, neural, and bone tumors with various imaging study modalities, including color duplex ultrasound and magnetic resonance imaging [6,7,9].

The available literature suggests that tibial nerve compression symptomatology will depend upon the degree of exerted compression, including motor and/or sensory involvement [9]. In this case, a 3x5 cm cyst mass provoked sensory symptoms with heel pain, swelling, paresthesias, hypoesthesia, and gastrocnemius muscle atrophy [8,9,12,13]. However, the incidence of tibial nerve compression (either by direct contact or by an increase of the intra-compartmental pressure) by a Baker's popliteal cyst is unknown due to underdiagnosis [8,13], and not even discussed among lower limb entrapment syndromes [14].

Asymptomatic Baker's cysts require no treatment, but surgical management should be considered when provoking pain and/or limiting knee range of motion [5,12]. In the present case report, surgical treatment was considered after the failure of a conservative approach, comprising pain medication, physiotherapy, and intra-articular corticosteroid injection. The surgical treatment included addressing the knee intra-articular lesions, ligation of the communication between the cyst and the articular cavity, and resection of the cyst wall, which is considered the classic management principles, with symptomatic resolution within one week after surgery. A systematic review by Zhou et al. pooled data from 11 studies, comparing different surgical methods in therapy of popliteal cyst, showing that following these principles yielded satisfactory outcomes in more than 95% of the patients, but data was insufficient to support arthroscopic over open approach [5]. Likewise, a retrospective study by Xinxian et al. including 65 patients with symptomatic popliteal cysts over eight years compared the arthroscopic management of intra-articular pathologies and decompression of the cyst with and without open cyst wall resection, revealing better clinical outcomes and lower recurrence rates when the latter is included, but with a longer surgical time and postoperative complications at a mean 33.3 months follow-up [15]. Although there was no recurrence during the one-year follow-up of the present case report, it represents the most common complication reported as high as in 63% of the cases [9,16].

In summary, the current case report comprises an uncommon presentation of symptomatic Baker's cyst, in which, after several months of knee pain and impaired range of motion, progressive sensory deficit and muscle atrophy without excessive muscle weakness were gradually established. Contrary to previously published reports, our patient did not have a history of rheumatoid diseases [9,10,17-19], degenerative lumbar spine disease [17,19], or motor deficit [12,17,18,20,21].

The present study has some limitations. Among them is the lack of nerve conduction studies, which can also aid in the suspicion of nerve compression and short-term follow-up [8,12,17]. However, it exposes the increasing relevance of standardized motor and sensory examination during routine follow-up of asymptomatic Baker's cysts, as it may help to improve the reporting quality and evidence of associated nerve compression of this condition. Furthermore, it points out that early diagnosis of associated nerve compression may result in better outcomes without long-term functional sequelae [20,21].

## Conclusions

Baker's cysts are common popliteal cystic tumors that could compromise the adjacent neurovascular structures and cause debilitating functional impairment. Surgical treatment yields satisfactory outcomes in patients who experience persistent pain, limited range of motion, and nerve compression symptoms in case conservative measures do not provide relief. This case report referred to a 46-year-old patient in which early diagnosis of a Baker's cyst with tibial nerve compression was done by observing sensory deficit. A combined arthroscopic treatment for intra-articular associated injuries and open cyst excision, after a conservative treatment failure, resulted in fast symptomatic relief and rapid return to sports and professional activities of the patient.
